# A PUBLIC AWARENESS MESSAGE TO DISRUPT AGEISM AND PREVENT ELDER ABUSE

**DOI:** 10.1093/geroni/igae098.0267

**Published:** 2024-12-31

**Authors:** Sarah Marrs

**Affiliations:** Virginia Commonwealth University, Richmond, Virginia, United States

## Abstract

To date, much of the research on elder mistreatment has focused on achieving improved skills for recognizing, screening for, and reporting abuse that has already occurred, typically considered secondary prevention. Knowing how to recognize when abuse has occurred is indeed critical for effective and timely intervention. However, engaging in efforts that challenge how we as a society perceive aging and older adults is an important and necessary primary prevention strategy, which targets stopping abuse from occurring in the first place by focusing on the source of abuse, such as underlying beliefs or attitudes. Given the linkages between ageism and abuse, our team has engaged in public education and awareness campaigns aimed at changing the narrative we as individuals associate with aging and older adults. In this multimedia presentation, we will begin by briefly discussing the link between ageism (both individual and structural) and elder abuse, citing current scientific literature as well as our own research, and how this led to our receipt of funding from the Administration for Community Living (ACL) to conduct innovative elder justice training and research. Then, we will briefly discuss the methods and evaluation feedback for one small component of this work, an ageism and abuse public awareness video. Specifically, we will highlight the methods (i.e., qualitative data collected, literature reviewed) that informed the creation of the video, present the video, and then share the feedback we have collected as part of our overall project evaluation activities.

